# Nurses' knowledge on pressure ulcer prevention: An updated systematic review and meta-analysis based on the Pressure Ulcer Knowledge Assessment Tool

**DOI:** 10.3389/fpubh.2022.964680

**Published:** 2022-09-08

**Authors:** Jing Wu, Bangjun Wang, Liping Zhu, Xiaoli Jia

**Affiliations:** ^1^Department of Pediatric Hematologic Oncology Nursing, West China Second University Hospital, Sichuan University, Chengdu, China; ^2^Key Laboratory of Birth Defects and Related Diseases of Women and Children, Ministry of Education, Sichuan University, Chengdu, China; ^3^Department of Obstetrics and Gynecology, Mianyang Central Hospital, Mianyang, China

**Keywords:** pressure ulcer, prevention knowledge, PUKAT, prevention, knowledge

## Abstract

**Background:**

Pressure ulcers (PUs) are an indicator of the quality of nursing care and nurses can prevent PUs well if they have sufficient knowledge. Numerous studies in this field have reported different results. The aim of this study was to estimate the pooled score of nurses' knowledge about PU prevention based on the Pressure Ulcer Knowledge Assessment Tool (PUKAT).

**Methods:**

In this systematic review and meta-analysis, databases including Web of Science, ScienceDirect, PubMed, and Scopus were searched. All studies published in English between 2011 and 2022 that reported the status of nurses' knowledge of PU prevention based on PUKAT were included in the analysis. Based on heterogeneity between the studies, the data were analyzed using a random effects model.

**Results:**

The pooled scores of PU prevention knowledge in nurses and nursing students were (51.5%; 95% CI: 45.8–57.2%) and (48.9%; 95% CI: 42.5–55.2%), respectively. As the age of the participants increased, the pooled score of pressure ulcer prevention knowledge increased significantly (*p* = 0.028). The publication bias was not significant. The highest and lowest knowledge scores in nurses and nursing students were related to the fourth dimension (nutrition) and the fifth dimension (preventive measures to reduce the amount of pressure/shear), respectively.

**Conclusion:**

Knowledge of nurses and nursing students about PU prevention is insufficient. Providing regular training to nurses and including the principles of PU prevention in the curriculum of nursing students to improve their knowledge seems necessary.

## Introduction

Pressure ulcer (PU) is a localized lesion on the skin and/or underlying tissue that often occurs on bony prominences due to pressure or pressure in combination with shear and/or friction forces (1). Most PUs are avoidable; however, the prevalence of PUs remains high (2). The burden of PUs is so high that some regulatory bodies have set goals to reduce the number of patients, and others have introduced financial penalties and/or incentives schemes to reduce the development of PUs (3, 4). The prevalence of PU is mentioned as an indicator of the quality of hospital care, which is widely accepted as a nursing-sensitive measure (5). In addition to causing suffering and reducing the quality of life of patients, PUs are associated with high costs of health care and prolonged nursing care (6, 7), and can lead to life-threatening situations (8).

Social exclusion, malodor, fluid leakage, pain, immobility, loss of independence, and changes in body image all affect the quality of life of these patients (9, 10). Nurses are responsible for providing safe prevention of PUs in at-risk patients (11), but it is often seen that they have poor adherence to pressure ulcer prevention guidelines, which may be due to their lack of knowledge (12). Sometimes nurses are not fully aware of the importance of using up-to-date PU prevention protocols or may not be exposed to current trends. Therefore, instead of using evidence-based methods, they take preventive measures based on intuition, experience, or habit (13).

PU prevention is very important because 95% of all PUs are preventable, therefore, nurses who work in clinical settings and are in daily contact with people at high risk for PU should have adequate knowledge level and a positive attitude (14, 15). Insufficient knowledge and skills in PU prevention can increase or exacerbate the chances of developing PUs, so nurses need regular training in this area (12). Improving nurses' knowledge of PU prevention not only improves the quality of PU care, but also reduces the length of hospital stay and the number of patients suffering from pressure ulcers (16). Knowledge of PU prevention helps nurses better decide which patients should receive prevention, which prevention should be applied, and how prevention must be applied (17).

Despite the importance of PU prevention and the development of international evidence-based guidelines, various studies on nurses' knowledge of risk assessment and PU prevention have shown different results. In a systematic review study, Dalvand et al. (18) examined nurses' knowledge of PU prevention. They reviewed eight eligible studies and concluded that the knowledge of nurses and nursing students in this field is still insufficient (18). Therefore, it is necessary to systematically review the results of all related studies and estimate the level of knowledge of nurses about pressure ulcer prevention. The aim of this updated systematic review and meta-analysis was to estimate the pooled score of nurses' knowledge about pressure ulcer prevention based on the Pressure Ulcer Knowledge Assessment Tool (PUKAT).

## Methods

In this systematic review and meta-analysis, the level of nurses' knowledge about PU prevention was estimated based on PUKAT and according to preferred reporting items for systematic reviews and meta-analyses (PRISMA) guidelines (19).

### Search strategy

In this study, the knowledge scores of nurses and nursing students were evaluated in articles published in English based on PUKAT. To search was conducted in PubMed, Scopus, ScienceDirect, and Web of Science using the following keywords: pressure ulcer, bedsore, pressure sore, decubitus ulcer, knowledge and all their possible combinations were searched. The references of the selected articles were also reviewed for access to other articles. Also, in Google Scholar, all the articles that cited the main article (development and validation of PUKAT) were reviewed. Considering that the PUKAT was developed and validated in 2011, databases were searched from 2011 to 2022. The search results of the mentioned databases are presented in Table 1.

**Table 1:** The result of search strategy.

PubMed	(“Pressure Ulcer”[Mesh] OR “Pressure Ulcer*”[tiab] OR “Bedsore*”[tiab] OR “Bed sore*”[tiab] OR “Pressure Sore*”[tiab] OR “Decubitus Ulcer*”[tiab]) AND (“Knowledge”[Mesh] OR “Awareness”[Mesh] OR “Knowledge”[tiab] OR “Epistemology”[tiab] OR “Awareness*”[tiab] OR “Situational Awareness*”[tiab] OR “Situation Awareness*”[tiab]) AND (“Nurses”[Mesh] OR “Nurse*”[tiab] OR “Nursing Personnel*”[tiab] OR “Registered Nurs*”[tiab] OR “Students, Nursing”[Mesh] OR “Nursing Staff”[Mesh] OR “Nursing Student*”[tiab] OR “Pupil Nurse*”[tiab] OR “Nursing Staff*”[tiab]) AND (“Pressure Ulcer Knowledge Assessment Tool”[all] OR “PUKAT*”[all])
Scopus	TITLE-ABS-KEY (“Pressure Ulcer*” OR “Bedsore*” OR “Bed sore*” OR “Pressure Sore*” OR “Decubitus Ulcer*”) AND TITLE-ABS-KEY (“Knowledge” OR “Epistemology” OR “Awareness*” OR “Situational Awareness*” OR “Situation Awareness*”) AND TITLE-ABS-KEY (“Nurse*” OR “Nursing Personnel*” OR “Registered Nurs*” OR “Nursing student*” OR “Pupil Nurse*” OR “Nursing Staff*”) AND ALL (“Pressure Ulcer Knowledge Assessment Tool” OR “PUKAT*”)
Web of Science	TS= (“Pressure Ulcer*” OR “Bedsore*” OR “Bed sore*” OR “Pressure Sore*” OR “Decubitus Ulcer*”) AND TS=(“Knowledge” OR “Epistemology” OR “Awareness*” OR “Situational Awareness*” OR “Situation Awareness*”) AND TS=(“Nurs*” OR “Nursing Personnel*” OR “Registered Nurse*” OR “Nursing student*” OR “Pupil Nurse*” OR “Nursing Staff*”) AND ALL=(“Pressure Ulcer Knowledge Assessment Tool” OR “PUKAT*”) Timespan: All years. Indexes: SCI-EXPANDED, SSCI, A&HCI, ESCI.
ScienceDirect	(“Pressure Ulcer” OR “Bedsore” OR “Bed sore” OR “Pressure Sore” OR “Decubitus Ulcer”) AND (“Knowledge” OR “Epistemology” OR “Awareness”) AND (“Nurse” OR “Nursing Staff” OR “Nursing student*”) AND (“Pressure Ulcer Knowledge Assessment Tool” OR “PUKAT”)

### Selection of studies and data extraction

Initially, the two researchers independently screened articles based on title and abstract, eliminating irrelevant studies. Then the full text of the remaining articles was read, and studies that did not report the required information were excluded from the analysis based on inclusion and exclusion criteria. The inclusion criteria were: conducting a study on nurses or nursing students, using PUKAT to measure the knowledge of pressure ulcer prevention, reporting the overall score or the score of different dimensions of knowledge, and publishing in English. Eligible articles were reviewed by two independent researchers and the necessary information such as the first author, year of publication, sample size, target group, total score of knowledge, and score of different dimensions of knowledge were extracted and recorded in the pre-prepared form. Studies that did not report essential information, whose full text was not available, or measured knowledge based on other tools were excluded from the analysis. In all stages of reviewing and evaluating the articles, any disagreements were resolved through consultation.

### The Pressure Ulcer Knowledge Assessment Tool

The PUKAT was developed by Beeckman et al. (17) and includes 26 questions and 6 dimensions: (1) etiology and development, (2) classification and observation, (3) nutrition, (4) risk assessment, (5) reduction of the magnitude of pressure and tearing, and (6) reduction of the duration of pressure and shearing. Each question has multiple answers, one of which is correct and the rest are incorrect. The correct answer is given a score of “1” and the incorrect answers are given a score of “zero”. The overall score and the score of each dimension are reported as a percentage. The final score varies between zero and 26, a higher score indicates more knowledge. Achieving more than 60% of the knowledge score indicates a sufficient level of knowledge (17).

### Quality assessment

The strengthening the reporting of observational studies in epidemiology (STROBE) checklist was used to evaluate the methodological quality of the selected articles. Based on the nature and purpose of the study, 10 items were selected from this checklist and selected articles were evaluated based on them. If those items were observed in the analyzed articles, they would be given a score of 1 and otherwise it would be given a score of zero. Therefore, the final score ranges from 0 to 10, and a higher score indicates a better quality (20). Based on the score of this checklist, the articles were divided into three categories: weak (score less than 4), medium (4 to 7), and strong (above 7).

### Statistical analysis

In these studies, the total knowledge score and its dimensions were expressed as a percentage, so we used a binomial distribution to estimate the pooled score and its dimensions. The I^2^ index and the Cochrane Q test were used to examine the heterogeneity between the selected studies. Considering that I^2^ index was more than 75% (*I*^2^ value > 75% is considered as high heterogeneity) and Cochran Q test was also significant (significance level for this test was considered 0.1), random effects model was used to combine selected studies and estimate the percentage of scores. Forrest Plot was used to visually display selected studies in terms of effect size and 95% confidence interval. Because nursing students' curricula may not have educational material on chronic wounds or may be limited, they could not be grouped with nurses who have experience working in the clinic and dealing with these wounds. Therefore, subgroup analysis was presented separately for the target groups (nursing students and nurses). Also, considering that in selected studies, the level of knowledge was measured based on two versions PUKAT 1 and PUKAT 2, we reported the results separately based on the version used.

Meta-regression analysis was used to examine the relationship between the year of publication, sample size and mean age of participants. To evaluate the effect of small studies and potential publication bias, funnel plot based on Egger regression test was used. All analyzes were performed using STATA software version 16.

## Results

**Figure 1:**
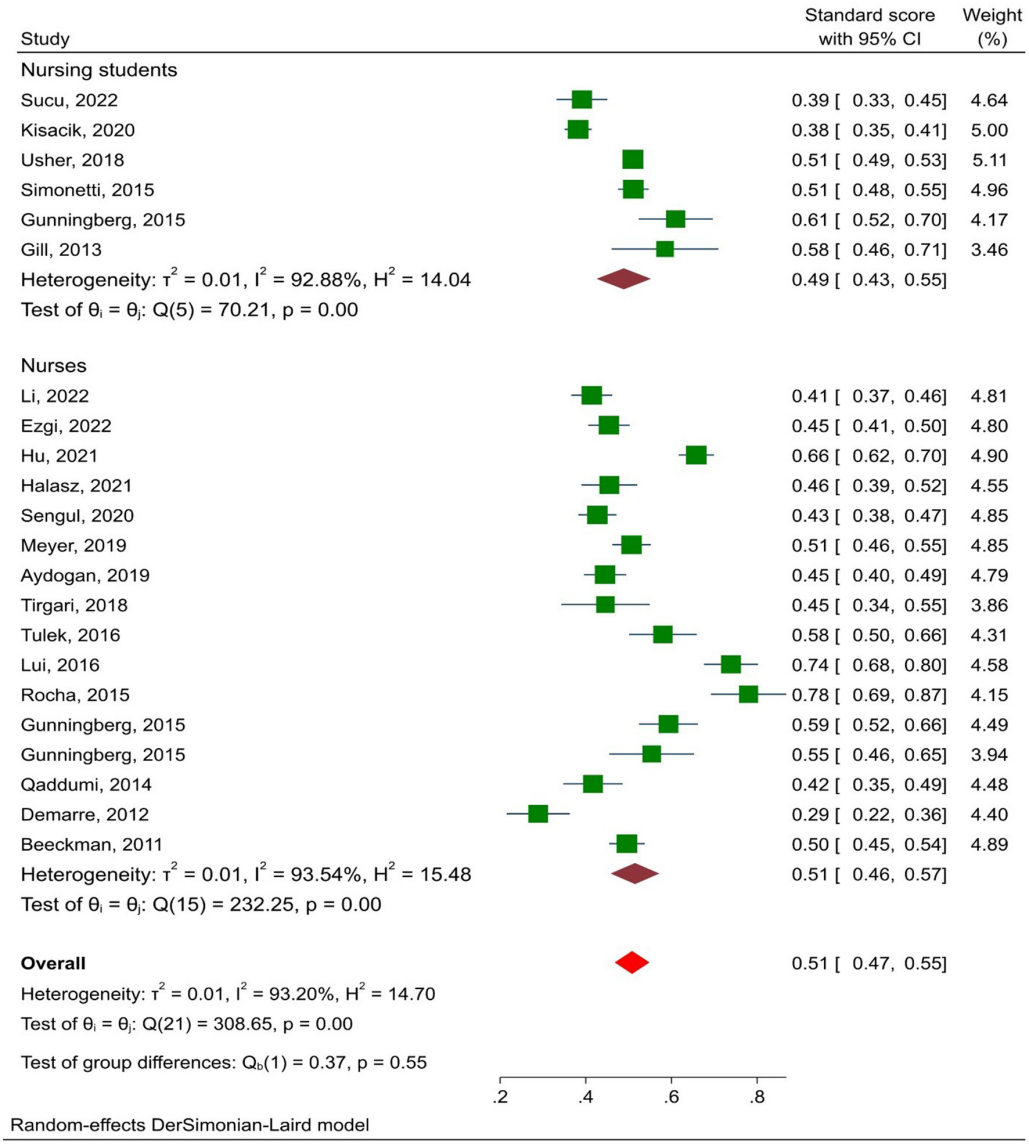
Screening flowchart showing the selection of qualified articles according to the PRISMA statement.

In this study, all published studies that examined the knowledge of nurses and nursing students about the prevention of pressure ulcers (based on PUKAT) were reviewed. Because the PUKAT was designed by Beckman et al., (17), studies between 2011 to 2022 were included in the analysis. In the initial search, 501 studies were identified and 481 studies were excluded from the final analysis based on inclusion and exclusion criteria (Figure 1).

In this systematic review, 20 eligible studies were included in the analysis. The studies were conducted between 2011 and 2022. The study of Gunningberg et al. was performed on nurses and nursing students and the details of the dimensional score were reported separately (2). Another 5 studies were performed on nursing students and 14 studies were performed on nurses. Most of the studies were related to Turkey (*n* = 6). The highest and lowest sample sizes were related to the studies of Usher et al. and Gill et al., respectively (21, 22). None of the selected articles had low methodological quality. More details are given in Table 2. PUKAT 2 was used in three articles, all of which were performed on nurses. Also, in all studies conducted on nursing students, the level of knowledge was measured based on PUKAT 1.

**Table 2:** The characteristics of selected studies.

**First author**	**Year**	**Sample size**	**Country**	**Target group**	**Score (%)**
Li et al. (23)	2022	404	Australia	Nurses	41.4
Dirgar et al. (24)	2022	406	Turkey	Nurses	45.4
Dag Sucu et al. (25)	2022	259	Turkey	Nursing students	39.1
Hu et al. (26)	2021	510	China	Nurses	65.8
Halasz et al. (8)	2021	225	Slovakia	Nurses	45.5
Sengul et al. (27)	2020	471	Turkey	Nurses	42.7
Kisacik et al. (28)	2020	908	Turkey	Nursing students	38.2
De Meyer et al. (29)	2019	474	Belgium	Nurses	50.7
Aydogan et al. (30)	2019	390	Turkey	Nurses	44.5
Tirgari et al. (31)	2018	89	Iran	Nurses	44.6
Usher et al. (22)	2018	2949	Australia	Nursing students	51
Tulek et al. (32)	2016	150	Turkey	Nurses	58
Lui et al. (10)	2016	186	China	Nurses	73.9
Rocha et al. (33)	2015	85	Brazil	Nurses	78
Simonetti et al. (34)	2015	742	Italy	Nursing students	51.1
Gunninberg et al. (2)	2015	122	Sweden	Nursing students	61
		196		Nurses	59.3
		97		Nurses	55.4
Qaddumi et al. (12)	2014	194	Jordan	Nurses	41.7
Gill et al. (21)	2013	60	Ireland	Nursing students	58.5
Demarre et al. (35)	2012	145	Belgium	Nurses	28.9
Beeckman et al. (36)	2011	553	Belgium	Nurses	49.6

**Figure 2:**
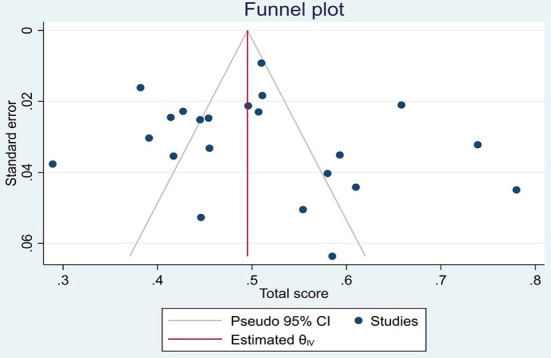
Forest plot showing the pooled scores on PU prevention based on the target group.

The pressure ulcer prevention knowledge score was 51% (95% CI: 47–55%). The results of subgroup analysis by target population showed that the knowledge scores of nurses and nursing students were (51.5%; 95% CI: 45.8–57.2%) and (48.9%; 95% CI: 42.5–55.2%), respectively (Figure 2). In three studies, the second version of the PUKAT was used, which had 28 questions instead of 26. The knowledge score based on the PUKAT 1 and PUKAT 2 was 50.5% (95% CI: 46.2–54.7%) and 52.7% (95% CI: 38.5–66.8%), respectively (*p* = 0.769). Also, nurses and nursing students did not have significant differences in any of the dimensions of PUKAT.

The highest score of pressure ulcer prevention knowledge in both groups of nurses (70%, 95% CI: 42–98) and nursing students (74.9%, 95% CI: 54.7–95.1) was related to the fourth dimension (nutrition). Nurses (45.7%, 95% CI: 36.8–54.5) and nursing students (40.2%, 95% CI: 35.8–44.6) also had the lowest scores in the fifth dimension (preventive measures to reduce the amount of pressure/shear) (Table 3).

**Table 3:** Scores of different dimensions of PU prevention by target group.

**Dimension**	**Target group**	**Score (95% CI)**	**Between studies**			**Between subgroups**	
			**I^2^**	**P _heterogeneity_**	**Q**	**Q**	**P _heterogeneity_**
D1	Nursing students	46.8 (36.5–57.1)	97.60	0.001	166.97	0.27	0.602
	Nurses	51.2 (38.2–64.2)	96.73	0.001	94.89		
D2	Nursing students	47.4 (40–54.9)	95.26	0.001	84.31	0.57	0.451
	Nurses	53.7 (39.2–68.1)	95.84	0.001	120.28		
D3	Nursing students	54.6(40.9–68.3)	99.37	0.001	474.09	0.08	0.779
	Nurses	65.1 (47.7–82.5)	99.41	0.001	851.37		
D4	Nursing students	74.9 (54.7–95.1)	98.72	0.001	312.68	0.86	0.354
	Nurses	70 (42–98)	97.69	0.001	216.05		
D5	Nursing students	40.2 (35.8–44.6)	85.77	0.001	28.12	1.18	0.278
	Nurses	45.7 (36.8–54.5)	88.19	0.001	42.34		
D6	Nursing students	48.6 (42.5–54.6)	92.60	0.001	54.04	1.12	0.290
	Nurses	54.4 (45.5–63.3)	88.23	0.001	42.50		
Total score	Nursing students	49 (43–55)	92.88	0.001	70.21	0.37	0.546
	Nurses	51 (46–57)	93.54	0.001	232.25		

Domain 1: Etiology and development; Domain 2: Classification and observation; Domain 3: Risk assessment; Domain 4: nutrition; Domain 5: Preventive measures to reduce the amount of pressure/shear; and Domain 6: Preventive measures to reduce the duration of pressure/shear.

**Figure 3:**
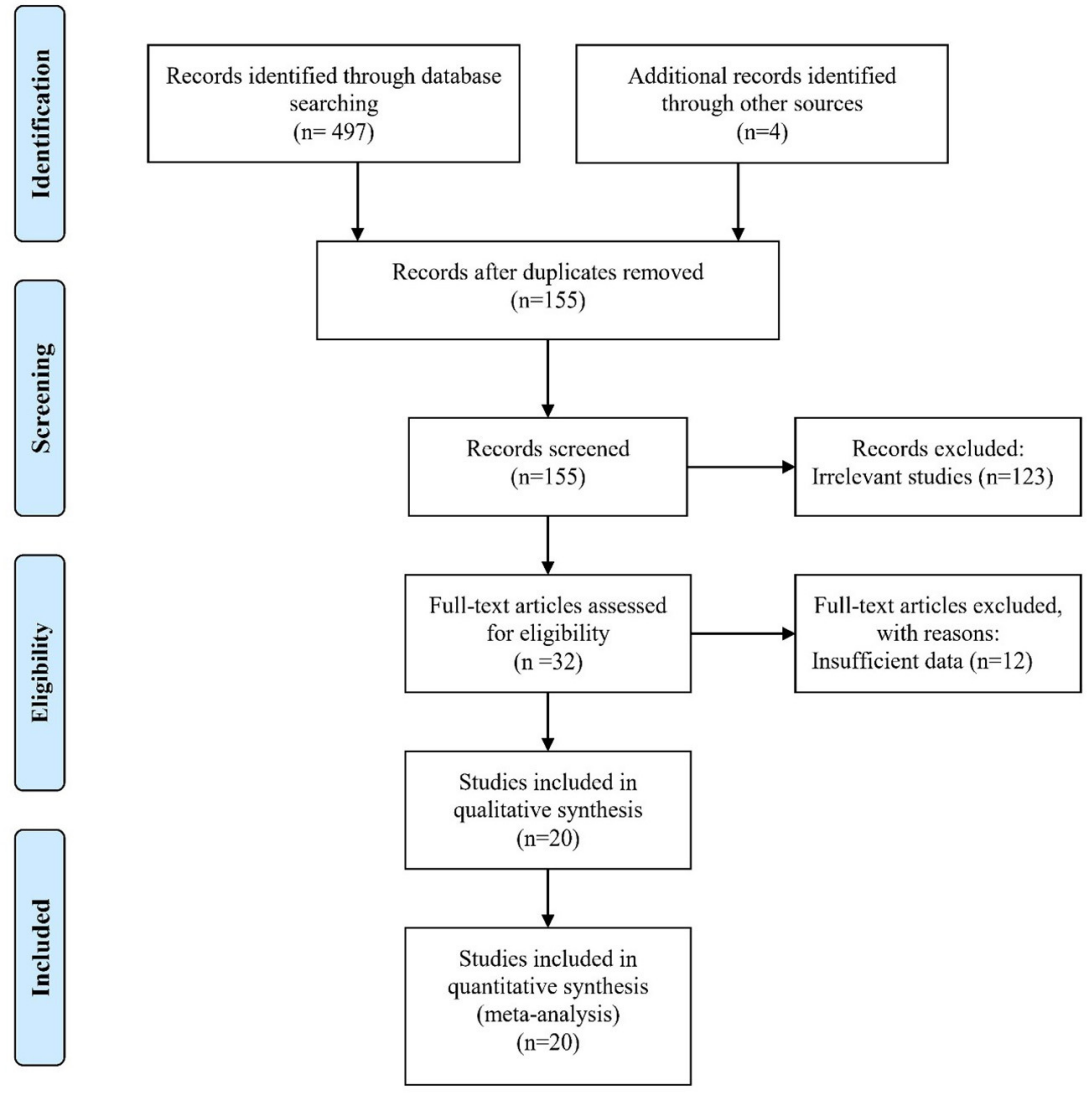
PRISMA 2019 flow diagram. Publication bias.

The results of meta-regression showed that there was no relationship between the score of PU prevention knowledge with the year of publication (*p* = 0.30) and sample size (*p* = 0.632), but with age, the knowledge of PU prevention increased significantly (*p* = 0.028). Also, publication bias was not significant (*p* = 0.220) (Figure 3).

## Discussion

The knowledge scores of nurses and nursing students were 51.5 and 48.9%, respectively. Considering that obtaining more than 60% of the score indicates appropriate knowledge, the results of this study show that the knowledge of nurses and nursing students about the prevention of PU is insufficient. In the systematic review of Dalvand et al. the knowledge of nurses and nursing students was 55.4 and 52.7%, respectively, which is consistent with the results of the present study and emphasizes that the knowledge of pressure ulcer prevention has not yet been sufficient (18). The results of a study showed that neither nurses nor nursing students knew that the main cause of PUs was lack of oxygen in the tissue (2). It seems that until nurses do not have clear information about the root cause of PUs, patients cannot hope to receive evidence-based prevention measures.

The highest score of knowledge in nurses and nursing students was related to nutrition, which is exactly in line with the results of meta-analysis of Dalvand et al. (18). This finding may be due to the small number of questions in this dimension or the simplicity of these questions compared to other questions. A recent systematic review and meta-analysis concluded that high-protein oral supplements reduced the effects of PUs and hospitalization (37). However, dietary supplements cannot replace pressure reduction to prevent PU. The lowest knowledge score in nurses and nursing students was related to the fifth dimension. This dimension of knowledge of nurses and nursing students refers to such things as changing positions, positions that reduce the risk of pressure ulcers, scheduling changes in the patient lying on visco-elastic foam, and the disadvantages of water mattresses and common sites of pressure ulcers. In the study by Schoeps et al., most nurses did not follow pressure ulcer prevention strategies such as changing positions (38). The results of the study by Mwebaza et al. showed that one-third of the nurses did not observe their bodies for the presence of pressure ulcers during the admission of patients, so the presence of pressure ulcers was ignored (39). In this study, with increasing age, knowledge score increased significantly. Given that the mean age of nurses is higher than nursing students, this finding is acceptable. Due to the lack of relationship between knowledge score and the year of publication of studies, it can be stated that between 2011 and 2022, the knowledge score of nurses and nursing students in the field of PU prevention has not changed significantly. One of the limitations of this study is that the assessment of nurses' knowledge based on PUKAT tools is only able to measure declarative knowledge and does not examine high levels of knowledge such as analysis, synthesis and evaluation. Another limitation of this study was the high heterogeneity, which indicates that the published studies are heterogeneous and not all conducted in the same direction. Therefore, it is suggested to conduct cohort studies with a large sample size in this field in the future.

## Conclusion

The results of this study showed that the knowledge of nurses and nursing students about the PUs prevention is insufficient and until the level of their knowledge reaches an acceptable level, the prevalence of PUS cannot be expected to decrease significantly. Accordingly, providing the necessary training to nurses and allocating part of the curriculum of nursing students to the principles of PU prevention can be helpful. Due to the low score of knowledge of both nurses and nursing students in the fifth dimension of PUKAT 1, providing the necessary explanations about “preventive measures to reduce the amount of pressure/shear” to improve this section will improve their overall knowledge.

## Data availability statement

The original contributions presented in the study are included in the article/supplementary material, further inquiries can be directed to the corresponding author/s.

## Author contributions

JW and BW extracted and analyzed the synthesized data and made a major contribution in writing the manuscript from overall and data analytical perspective. LZ and XJ interpreted data. All authors read and approved the final manuscript.

## Conflict of interest

The authors declare that the research was conducted in the absence of any commercial or financial relationships that could be construed as a potential conflict of interest.

## Publisher's note

All claims expressed in this article are solely those of the authors and do not necessarily represent those of their affiliated organizations, or those of the publisher, the editors and the reviewers. Any product that may be evaluated in this article, or claim that may be made by its manufacturer, is not guaranteed or endorsed by the publisher.

## Abbreviations

PRISMA: Preferred reporting items for systematic reviews and meta-analyses
PU: Pressure ulcers
PUKAT: Pressure Ulcer Knowledge Assessment Tool
STROBE: The strengthening the reporting of observational studies in epidemiology.
